# Serum neurofilament as a predictor of 10-year grey matter atrophy and clinical disability in multiple sclerosis: a longitudinal study

**DOI:** 10.1136/jnnp-2021-328568

**Published:** 2022-06-01

**Authors:** Ingrid Anne Lie, Sezgi Kaçar, Kristin Wesnes, Iman Brouwer, Silje S Kvistad, Stig Wergeland, Trygve Holmøy, Rune Midgard, Alla Bru, Astrid Edland, Randi Eikeland, Sonia Gosal, Hanne F Harbo, Grethe Kleveland, Yvonne S Sørenes, Nina Øksendal, Kristin N Varhaug, Christian A Vedeler, Frederik Barkhof, Charlotte E Teunissen, Lars Bø, Øivind Torkildsen, Kjell-Morten Myhr, Hugo Vrenken

**Affiliations:** 1 Department of Clinical Medicine, University of Bergen, Bergen, Norway; 2 Neuro-SysMed, Department of Neurology, Haukeland University Hospital, Bergen, Norway; 3 Department of Radiology and Nuclear Medicine, MS Center Amsterdam, Amsterdam Neuroscience, Amsterdam UMC, Amsterdam, The Netherlands; 4 Department of Neurology, St. Olav's University Hospital, Trondheim, Norway; 5 Department of Immunology and Transfusion Medicine, Haukeland University Hospital, Bergen, Norway; 6 Norwegian Multiple Sclerosis Registry and Biobank, Department of Neurology, Haukeland University Hospital, Bergen, Norway; 7 Institute of Clinical Medicine, University of Oslo, Oslo, Norway; 8 Department of Neurology, Akershus University Hospital, Lorenskog, Norway; 9 Department of Neurology, Molde Hospital, Molde, Norway; 10 Department of Neurology, Stavanger University Hospital, Stavanger, Norway; 11 Department of Neurology, Vestre Viken Hospital Trust, Drammen, Norway; 12 Department of Research and Education, Sørlandet Hospital Trust, Kristiansand, Norway; 13 Faculty of Health and Sport Science, University of Agder, Grimstad, Norway; 14 Department of Neurology, Østfold Hospital Kalnes, Grålum, Norway; 15 Department of Neurology, Oslo University Hospital, Oslo, Norway; 16 Department of Neurology, Innlandet Hospital Trust, Lillehammer, Norway; 17 Department of Neurology, Haugesund Hospital, Haugesund, Norway; 18 Department of Neurology, Nordland Hospital Trust, Bodø, Norway; 19 Institutes of Neurology and Healthcare Engineering, UCL, London, UK; 20 Neurochemistry Laboratory, Clinical Chemistry Department, Amsterdam Neuroscience, Amsterdam University Medical Centres, Amsterdam, The Netherlands; 21 Norwegian Multiple Sclerosis Competence Centre, Department of Neurology, Haukeland University Hospital, Bergen, Norway

**Keywords:** MULTIPLE SCLEROSIS, CLINICAL NEUROLOGY, BIOCHEMISTRY, MRI

## Abstract

**Background:**

The predictive value of serum neurofilament light chain (sNfL) on long-term prognosis in multiple sclerosis (MS) is still unclear.

**Objective:**

Investigate the relation between sNfL levels over a 2-year period in patients with relapsing-remitting MS, and clinical disability and grey matter (GM) atrophy after 10 years.

**Methods:**

85 patients, originally enrolled in a multicentre, randomised trial of ω−3 fatty acids, participated in a 10-year follow-up visit. sNfL levels were measured by Simoa quarterly until month 12, and then at month 24. The appearance of new gadolinium-enhancing (Gd+) lesions was assessed monthly between baseline and month 9, and then at months 12 and 24. At the 10-year follow-up visit, brain atrophy measures were obtained using FreeSurfer.

**Results:**

Higher mean sNfL levels during early periods of active inflammation (Gd+ lesions present or recently present) predicted lower total (β=−0.399, p=0.040) and deep (β=−0.556, p=0.010) GM volume, lower mean cortical thickness (β=−0.581, p=0.010) and higher T2 lesion count (β=0.498, p=0.018). Of the clinical outcomes, higher inflammatory sNfL levels were associated with higher disability measured by the dominant hand Nine-Hole Peg Test (β=0.593, p=0.004). Mean sNfL levels during periods of remission (no Gd+ lesions present or recently present) did not predict GM atrophy or disability progression.

**Conclusion:**

Higher sNfL levels during periods of active inflammation predicted more GM atrophy and specific aspects of clinical disability 10 years later. The findings suggest that subsequent long-term GM atrophy is mainly due to neuroaxonal degradation within new lesions.

**WHAT IS ALREADY KNOWN ON THIS TOPIC**

- There is increasing evidence to support the use of serum neurofilament light chain (sNfL), as a marker of acute inflammatory axonal damage, to monitor short-term disease activity, treatment response and disability progression in multiple sclerosis (MS). However, whether sNfL levels also predict disease progression and neurodegeneration over several years, and even decades, is less clear.

**WHAT THIS STUDY ADDS**

- We found that higher sNfL levels measured during periods of active inflammation predicted lower total grey matter (GM) volume, deep GM volume and cortical thickness and higher T2 lesion count after 10 years in patients with relapsing-remitting MS (RRMS). Higher sNfL levels were also associated with higher disability measured by the dominant hand Nine-Hole Peg Test.

**HOW THIS STUDY MIGHT AFFECT RESEARCH, PRACTICE AND/OR POLICY**

- As long-term atrophy progression in patients with RRMS seems to be driven by focal inflammatory damage, measuring sNfL levels during relapses may be a way to quantify the extent of ongoing axonal injury, possibly indicating the risk of future disease progression. This added information may support clinicians in subsequent monitoring and treatment decisions.

## Introduction

The pathological mechanisms in multiple sclerosis (MS) are highly complex, affecting both white matter (WM) and grey matter (GM) structures throughout the central nervous system.^1^ Inflammatory and neurodegenerative processes both seem to play a role in disease progression and disability accumulation,^2–4^ but there is large variability between patients and disease phenotypes.^4^ This pathophysiological and clinical heterogeneity underlines the need for robust biomarkers predicting future clinical disability. At the same time, this heterogeneity poses a challenge in developing such markers, as they should reliably capture and differentiate the various ongoing disease processes.^5^

Neurofilaments are proposed candidate biomarkers, reflecting axonal injury.^6^ These proteins are major components of the axonal cytoskeleton and are released into the extracellular fluid when neuroaxonal damage occurs.^6^ The neurofilament protein consists of multiple, differently sized subunits, of which the neurofilament light chain (NfL) assay is the most widely researched.^7^ NfL levels can be determined in blood serum or plasma, and serum NfL (sNfL) levels strongly correlate with CSF NfL levels.^8^ The suggested dynamic equilibrium between the two body fluids makes NfL a candidate biomarker, because reliable measurements can be obtained by venepuncture, rather than the more invasive lumbar puncture.

Elevated sNfL levels have been shown to reflect acute axonal damage during active inflammation,^9^ and increasing evidence support the use of sNfL to monitor short-term disease activity, treatment response and disability progression.^10^ Whether sNfL levels also predict disease progression and neurodegeneration over several years, and even decades, is less clear.^6 10–12^ Associations between sNfL and long-term disability progression are not consistent,^13 14^ and although some studies have found higher sNfL levels to be associated with brain^13 15 16^ and GM atrophy,^17–19^ studies with extensive follow-up time are few, especially studies considering GM atrophy.^17 19^ Clarifying the properties of NfL as a predictor of long-term neurodegeneration is further complicated by the dynamic nature of MS pathophysiological processes: elevated NfL levels during periods with active inflammation mainly reflect the extent of ongoing acute axonal damage, rather than any simultaneous neurodegenerative processes.^20^ Furthermore, inflammatory activity and axonal damage persist several months after the appearance of a gadolinium-enhancing (Gd+) lesion, causing a prolonged elevation of the NfL level.^9^ If and how this variability affects the relation between NfL levels and long-term future disability and brain atrophy is not clear.^12 17^ As one patient with relapsing-remitting MS (RRMS) may experience periods of both remission and active inflammation, attempts to separate and explore the predictive value of sNfL levels during these periods may clarify pathophysiological disease mechanisms, and be of clinical relevance (eg, deciding optimal timepoints for sNfL measurements). By separately analysing sNfL levels obtained during, and outside of episodes of evident inflammatory activity (ie, Gd+ lesions) over a 2-year period, the present study aims to investigate how periods of acute disease activity compare to more silent periods in RRMS in predicting clinical disability and GM atrophy, measured after 10 years.

## Materials and methods

### Participants

The included patients originally participated in a multicentre trial of ω−3 fatty acids in MS (the OFAMS Study), which has previously been described in detail.^21^

In the trial, 92 patients with RRMS were followed over 24 months, for the first 6 months randomised to either ω−3 fatty acids monotherapy or placebo. Starting at 6 months, both treatment groups received additional treatment with subcutaneously administered interferon beta-1a, 44 µg, three times weekly for the remaining 18 months of the trial. Patients attended regular follow-up visits for biochemical, radiological and clinical examinations, including the Expanded Disability Status Scale (EDSS), timed 25-foot walk test (T25FW), the dominant and non-dominant hand Nine-Hole Peg Test (D9-HPT and ND9-HPT) and the Paced Auditory Serial Addition Test (PASAT). All available patients in the OFAMS Study were invited to a 10-year follow-up visit, of which 85 (92%) accepted.^22^ All biochemical, radiological and clinical examinations from the OFAMS Study were repeated at their local study site, with the addition of the oral Symbol Digit Modalities Test (SDMT). Between the OFAMS Study and the 10-year follow-up visit, the participants had received treatment and monitoring as advised by their treating neurologist as part of routine care.

### Serum sampling and analysis

Serum samples collected during the OFAMS Study were stored at −80°C. As previously described,^23^ sNfL levels were measured in duplicates, from samples collected at baseline (BL) and at months 3, 6, 9, 12 and 24, using a Simoa assay and according to the manufacturer’s instruction (Quanterix, Billerica, USA).

### MRI data and analysis

#### The OFAMS Study

During the trial, patients underwent MRI imaging at BL, monthly for the first 9 months, and thereafter at month 12 and 24. MRI was performed at each study site using a 1.5 Tesla (T) MRI scanner with the standard head coil. After intravenous injection of gadolinium-based contrast agent, the imaging protocol included a 2D sagittal fluid-attenuated inversion recovery (FLAIR) (resolution: 0.98×0.98×1 mm^3^, echo time (TE)/repetition time (TR)=100/6000–10000 ms, number of excitations (NEX) 2, slice thickness 4 mm), 2D axial T1-weighted images (resolution: 0.49×0.49×1 mm^3^, TE/TR=10–20/500-750 ms, NEX 2, slice thickness 4 mm) as well as sagittal 3D T1-weighted spoiled gradient echo (Fast Field Echo (FFE)/Fast Low Angle Shot (FLASH)) images (resolution: 0.98×0.98×1 mm^3^, TE/TR=4.6/20 ms, flip angle 25°, NEX 1, slice thickness 1 mm).

Blinded assessment of the T2 and Gd+ lesion count (LC) at BL, and the appearance of new Gd+ lesions was conducted by two experienced neuroradiologists.

#### The 10-year follow-up visit

Imaging was performed at the different study sites, on a 3T MRI scanner if available, alternatively using a 1.5 T MRI scanner, with a standard head coil. The following MRI sequences were acquired: a T2-weighted 3D sagittal FLAIR (resolution: 1×1×1 mm^3^, TE/TR/inversion time (TI)=386/5000/1.65–2.2 ms) and a postcontrast T1-weighted 3D sagittal magnetization prepared rapid gradient echo sequence (resolution: 1×1×1 mm^3^, TE/TR/TI=2.28/1800/900 ms, flip angle 8°).

#### Lesion segmentation and morphological reconstruction

A detailed description of these methods has recently been described^24^ and is available in online supplemental appendix 1. Briefly, on images obtained at the 10-year follow-up visit, lesion segmentation was done on FLAIR images using Lesion Segmentation Tool (V.2.0.15; http://applied-statistics.de/lst.html),^25^ and morphological reconstruction was performed with FreeSurfer (V.7.1.1; http://surfer.nmr.mgh.harvard.edu/) on T1-weighted images.

10.1136/jnnp-2021-328568.supp1 Supplementary data

### Calculation of sNfL levels

**Figure 1:**
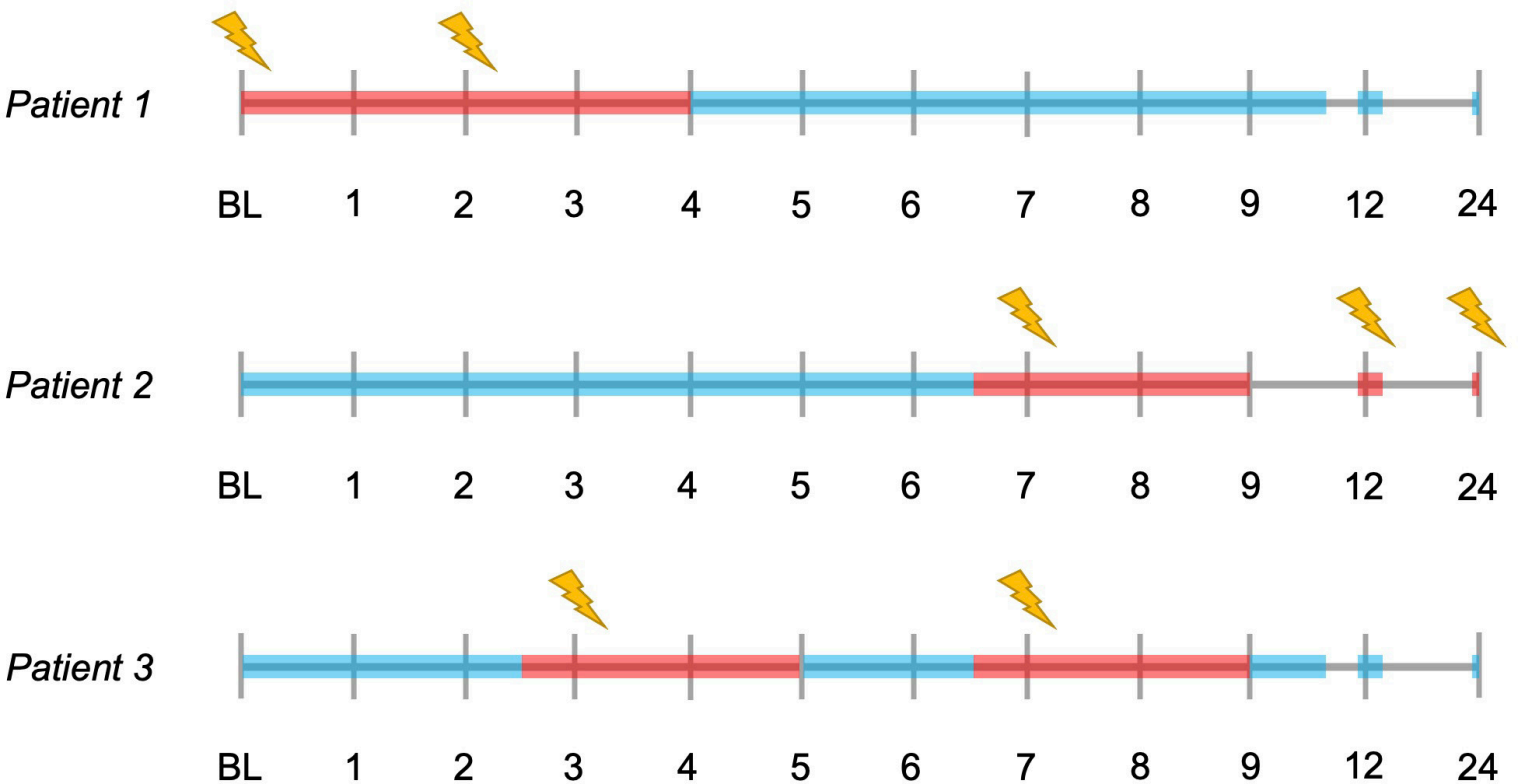
Illustrated examples of time periods where the collected serum neurofilament light chain (sNfL) levels are defined as ‘inflammatory’ or ‘non-inflammatory’. The timelines represent the MRI visits during the OFAMS Study; visits with a new gadolinium-enhancing lesion are marked with a lightning symbol. sNfL levels collected during periods marked in red are defined as inflammatory and levels collected during periods marked in blue are defined as non-inflammatory. With sNfL levels collected approximately at baseline, month 3, 6, 9, 12 and 24; patient 1 has two inflammatory (included in the analysis requiring at least two measurements, excluded from the analysis requiring at least three measurements) and four non-inflammatory sNfL levels (included in both analyses); patient 2 has three inflammatory (included in both analyses) and three non-inflammatory sNfL levels (included in both analyses); and patient 3 has two inflammatory (included in one analysis) and four non-inflammatory (included in both analyses) sNfL levels.

Mean sNfL levels were calculated, for each patient, for three different settings: ‘overall mean sNfL level’, from all samples collected between BL and month 24; ‘mean inflammatory sNfL level’, from samples collected within 2 months after the presence of a Gd+ lesion, or less than 2 weeks before the appearance of a Gd+ lesion (if collected more than 1 week after last MRI scan); and ‘mean non-inflammatory sNfL level’, from samples collected more than 2 months after the appearance of a Gd+ lesion and more than 2 weeks before the appearance of a new Gd+ lesion (if collected more than 1 week after last MRI scan). Examples of sNfL measurements defined as inflammatory and non-inflammatory are visualised in figure 1. In each patient, the mean inflammatory and non-inflammatory sNfL level was calculated separately for (1) at least two and (2) at least three measurements, when available. Measurements defined as inflammatory or non-inflammatory did not have to be collected at consecutive timepoints. The findings presented here were obtained using the mean of at least three measurements, highly comparable findings using the mean of at least two measurements are presented in the online supplemental tables 1 and 2.

10.1136/jnnp-2021-328568.supp2 Supplementary data

### Statistical analysis

Statistical analyses were performed using R software (V.4.0.5). Thalamus volume and mean cortical thickness in the left and right hemisphere were averaged.

To correct for the different study sites and scanner variability, the relationship between overall mean sNfL level and clinical and MRI atrophy measures was investigated by a linear multilevel regression model, corrected for age, sex, disease modifying therapy (DMT) use, estimated total intracranial volume (eTIV) (eTIV only included in analyses regarding MRI volume measures), fraction of MRI scans with new Gd+ lesions (fGd+), BL T2 and Gd+ LC, with study site entered as a random effect.

Between the OFAMS Study and the 10-year follow-up visit, patients underwent therapeutic interventions that varied both between and within patients, in potency, duration and time. A nominal variable was created based on the category (similar to those proposed in a recent study^26^) of DMT(s) used during the follow-up: (1) only used platform compounds (interferon beta and glatiramer acetate preparations), (2) ever used oral therapies (teriflunomide, dimethyl fumarate, fingolimod) and (3) ever used high efficiency monoclonal antibody therapies, chemotherapies or haematopoietic stem cell therapy.

For the relation between mean inflammatory and non-inflammatory sNfL levels and clinical and MRI atrophy measures, linear regression models were used, as entering the study site as a random effect did not improve the model. The first model (model 1) included mean inflammatory sNfL level, fGd+, age, sex, DMT use, eTIV, BL T2 and Gd+ LC as independent variables; the second model (model 2) included non-inflammatory sNfL level, age, sex, DMT use, eTIV, BL T2 and Gd+ LC. Lastly, a modified version of model 1 was used in two exploratory analyses: the first with the mean cortical thickness in the precentral gyrus as the dependent variable, and the second including MRI atrophy measures obtained at month 24 (available in a subset of patients) as a covariate. All independent variables were first entered as covariates and removed by backward elimination if not significant to the model. In case of missing observations, patients were excluded from the respective analyses. Assumptions for linear regression were checked for each final model; if the assumptions were not satisfied, log-linear transformation was performed (eg, logT25FW). The outcome measure EDSS≥4 was investigated by logistic regression. Lastly, the Benjamini-Hochberg method^27^ was used to control the false discovery rate (FDR) for multiple hypothesis testing. FDR controlling was performed for the main predictors (overall sNfL, inflammatory sNfL, non-inflammatory sNfL and fGd+) separately, including analyses with both MRI and clinical outcome measures.

## Results

### Patient characteristics

Of the 85 patients who participated in the 10-year follow-up visit, 78 had serum samples available for sNfL measurement and were included in this study. The mean follow-up time from BL to the 10-year follow-up visit was 12.0 years (±0.6). Table 1 summarises clinical and MRI characteristics of the included patients.

**Table 1:** Demographic, clinical and radiological characteristics

	N	Baseline	Month 24	10-year follow-up visit
Age in years, mean (SD)/median (range)	78			50.05 (8.4)/50.0 (31–70)
Sex, female, N (%)	78	51 (65.4%)		
Time since diagnosis, mean in years (SD)/median (range)	78			14.6 (3.4)/13.7 (11.0–26.1)
Disease phenotype (N)	78	RRMS (78)	RRMS (78)	RRMS (71), SPMS (7)
Type of DMT used during follow-up (N)	78	Only platform compounds* (23), ever used oral therapies† (32), ever used high efficiency monoclonal antibody therapies, chemotherapies, or HSCT‡ (23).		
Study site (number of patients)	78	Site 1 (3), site 2 (16), site 3 (3), site 4 (2), site 5 (1), site 6 (5), site 7 (8), site 8 (13), site 9 (3), site 10 (6), site 11 (2), site 12 (12), site 13 (4).		
EDSS, mean (SD)/median (range)	78/76/77	1.9 (0.8)/2.0 (0.0–4.0)	2.1 (1.2)/2.0 (0.0–5.0)	2.8 (1.6)/2.5 (0.0–8.5)
Mean sNfL level§ (pg/mL), mean (SD)	78	34.8 (14.3)		
Mean inflammatory sNfL level§ (pg/ml), mean (SD)	32	45.5 (21.3)		
Mean non-inflammatory sNfL level§ (pg/mL), mean (SD)	40	30.2 (9.5)		
fGd+, mean (SD)	78	0.32 (0.26)		
Number of MRI scans with new Gd-enhancing lesions, mean (SD)/median (range)	78	3.7 (3.1)/3.0 (0–11)		
Total GM volume (mm^3^), mean (SD)	65			630 134.461 (52 453.119)
Total WM volume (mm^3^), mean (SD)	65			448 155.938 (50 676.88)
Total deep GM volume (mm^3^), mean (SD)	65			55 726.031 (5291.634)
Thalamus volume (mm^3^), mean (SD)	65			7786.642 (982.467)
Mean Cth (mm), mean (SD)	65			2.538 (0.128)

*Interferon beta and glatiramer acetate preparations.

†Dimethyl fumarate, teriflunomide, fingolimod.

‡Natalizumab, rituximab, alemtuzumab, mitoxantrone, haematopoietic stem cell therapy.

§Mean sNfL levels measured from serum samples collected from baseline to month 24.

Cth, cortical thickness; DMT, disease modifying therapy; EDSS, Expanded Disability Status Scale; fGd+, fraction of MRI scans with new Gadolinium-enhancing lesion; Gd, Gadolinium; GM, grey matter; HSCT, haematopoietic stem cell therapy; RRMS, relapsing-remitting multiple sclerosis; sNfL, serum neurofilament light; SPMS, secondary progressive multiple sclerosis; WM, white matter.

### Overall mean sNfL level

Overall mean sNfL level did not predict any long-term MRI or clinical outcome measures, or change in clinical measures from month 24 to the 10-year follow-up (table 2).

**Table 2:** The association of overall mean sNfL level with MRI atrophy and clinical measures at the 10-year follow-up, with a random intercept for study site, corrected for age, sex, DMT use, eTIV, BL T2LC, BL Gd+ LC and fGd+

MRI/clinical measurement	N	B	Std. B	95% CI	P value*	Marginal R^2^	Conditional R^2^
Total GM volume	65	−471.6	−0.147	−1236.446 to 293.239	0.514	0.385	0.607
Total WM volume	65	−110.9	−0.030	−945.240 to 723.354	0.920	0.380	0.380
Total deep GM volume	65	−78.12	−0.221	−162.299 to 6.054	0.429	0.423	0.513
Thalamus volume	65	−12.778	−0.203	−29.365 to 3.808	0.487	0.276	0.501
Mean Cth	65	−0.002	−0.255	−0.004 to 1.069×10^−4^	0.782	0.308	0.584
logLesion volume†	68	−2.830×10^−4^	−0.001	−0.006 to 0.006	0.989	0.351	0.499
Lesion count	68	0.112	0.086	−0.046 to 0.270	0.488	0.272	0.430
EDSS≥4‡	77	0.000	1.000	0.952 to 1.052	0.985		
logT25FW†	72	−0.001	−0.158	−0.004 to 0.001	0.470	0.096	0.373
logChange in T25FW†	70	−0.001	−0.129	−0.003 to 3.932×10^−4^	0.581	0.062	0.258
logD9-HPT†	71	0.002	0.263	−4.058×10^−6^ to 0.004	1.000	0.309	0.348
logChange in D9-HPT†	69	−0.001	−0.076	−0.005 to 0.003	0.735	0.195	0.229
logND9-HPT†	70	−0.001	−0.073	−0.002 to 0.001	0.670	0.278	0.368
logChange in ND9-HPT†	68	−0.004	−0.234	−0.009 to 3.350×10^−4^	0.550	0.170	0.239
PASAT	72	0.088	0.112	−0.085 to 0.260	0.550	0.189	0.247
Change in PASAT	70	0.038	0.063	−0.082 to 0.157	0.738	0.143	0.431
Oral SDMT	67	0.110	0.128	−0.094 to 0.314	0.563	0.222	0.434

Marginal R^2^: variance explained by fixed effects.

Conditional R^2^: variance explained by both fixed and random effects.

*Adjusted p values after controlling the false discovery rate (FDR) for multiple hypothesis testing.

†Dependent variable log transformed due to non-normality (log-linear transformation).

‡Analysed by logistic regression, regression coefficient (B), odds ratio (Std. B) and 95% CI of odds ratio reported.

B, beta; BL, baseline; Cth, cortical thickness; D9-HPT, dominant hand Nine-Hole Peg Test; DMT, disease modifying therapy; EDSS, Expanded Disability Status Scale; eTIV, estimated total intracranial volume; fGd+, fraction of MRI scans with new Gadolinium-enhancing lesion; Gd+, gadolinium-enhancing; GM, grey matter; LC, lesion count; ND9-HPT, non-dominant hand Nine-Hole Peg Test; PASAT, Paced Auditory Serial Addition Test; SDMT, Symbol Digit Modalities Test; sNfL, serum neurofilament light chain; Std, standardised; T25FW, timed 25-foot walk; WM, white matter.

### Mean inflammatory sNfL level

The results of the linear regression model including inflammatory sNfL and fGd+ as predictor variables (model 1) are shown in table 3.

**Table 3:** Model 1: The association of inflammatory sNfL level and fGd+ with MRI atrophy and clinical measures at the 10-year follow-up, corrected for age, sex, DMT use, eTIV, BL T2LC and Gd+ LC*

		Mean inflammatory sNfL level				fGd+				Full model	
MRI/clinical measure	N	B	Std. B	95% CI	P value†	B	Std. B	95% CI	P value†	R^2^ adj.	P value
Total GM volume	25	−850.8	−0.399	−1580.218 to –121.416	**0.040**	91 552.9	0.362	3400.111 to 179 705.771	0.065	0.504	<0.001
Total WM volume	25				NS				NS		
Total deep GM volume	25	−140.31	−0.556	−228.417 to −52.198	**0.010**				NS	0.341	0.004
Thalamus volume	25				NS				NS		
Mean Cth	25	−0.003	−0.581	−0.005 to −0.001	**0.010**				NS	0.308	0.002
logLesion volume‡	28				NS				NS		
logLesion count‡	28	0.004	0.498	0.001 to 0.007	**0.018**				NS	0.220	0.007
EDSS≥4§	31				NS				NS		
logT25FW‡	30				NS				NS		
logChange in T25FW‡	30				NS				NS		
logD9-HPT‡	29	0.004	0.593	0.002 to 0.006	**0.004**				NS	0.411	0.001
logChange in D9-HPT‡	29				NS				NS		
logND9-HPT‡	29				NS				NS		
logChange in ND9-HPT‡	29	−0.006	−0.498	−0.010 to −0.001	**0.024**				NS	0.399	0.002
PASAT	28				NS				NS		
Change in PASAT	28				NS				NS		
Oral SDMT	28				NS				NS		

*Non-significant covariates removed from final model by backward elimination.

†Adjusted p values after controlling the false discovery rate (FDR) for multiple hypothesis testing.

‡Dependent variable log transformed due to non-normality (log-linear transformation).

§Analysed by logistic regression.

adj, adjusted; B, beta; BL, baseline; Cth, cortical thickness; D9-HPT, dominant hand Nine-Hole Peg Test; DMT, disease modifying therapy; EDSS, Expanded Disability Status Scale; eTIV, estimated total intracranial volume; fGd+, fraction of MRI scans with new Gadolinium-enhancing lesion; Gd+, gadolinium-enhancing; GM, grey matter; LC, lesion count; ND9-HPT, non-dominant hand Nine-Hole Peg Test; PASAT, Paced Auditory Serial Addition Test; SDMT, Symbol Digit Modalities Test; sNfL, serum neurofilament light chain; Std, standardised; T25FW, timed 25-foot walk; WM, white matter.

**Figure 2:**
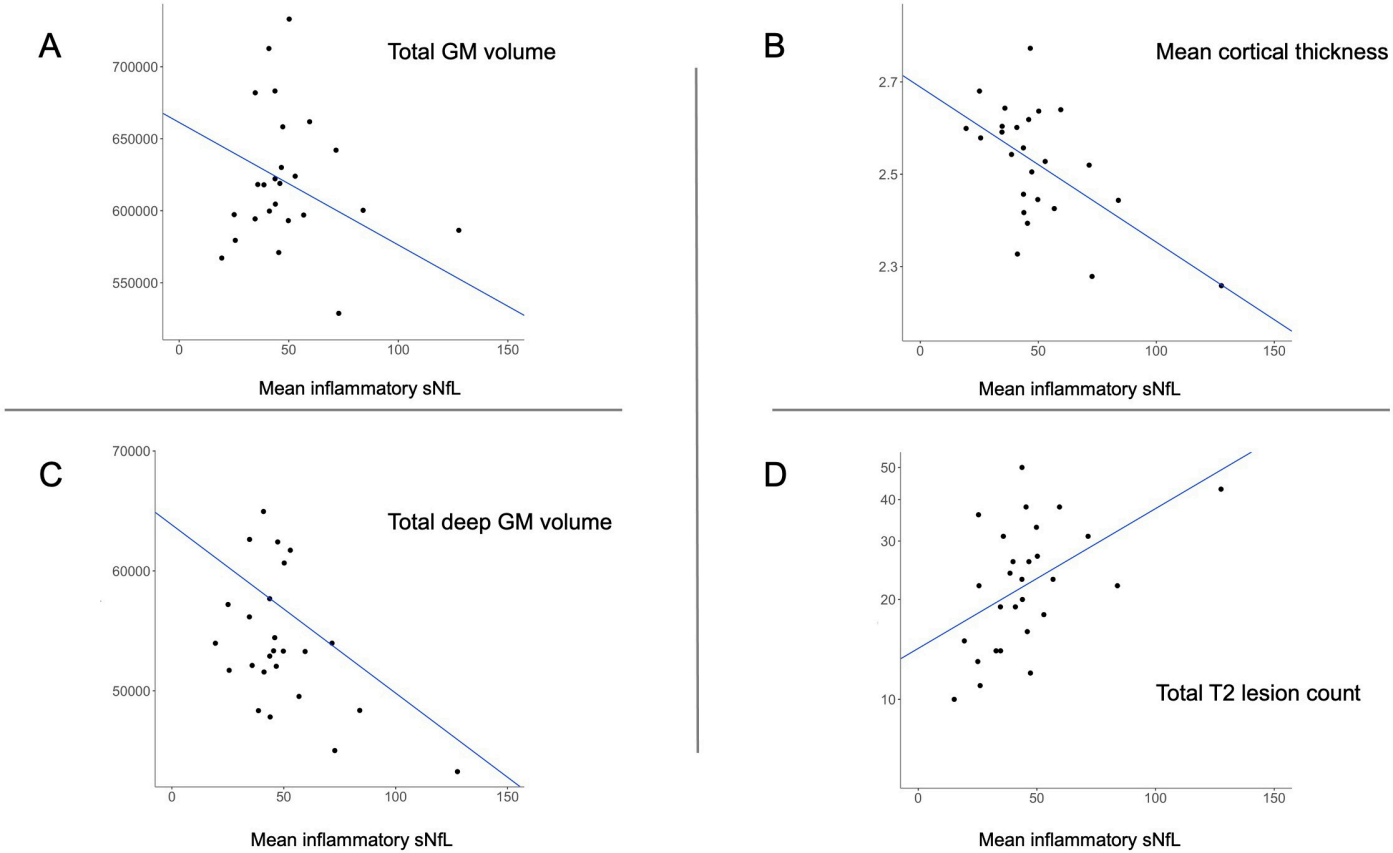
Scatterplots illustrating significant associations between mean inflammatory sNfL level (pg/mL) and (A) total GM volume (mm^3^), (B) mean cortical thickness (mm), (C) total deep GM volume (mm^3^) and (D) total T2 lesion count (N). The Y-axis is transformed to logarithmic scale to illustrate the absolute lesion count. Cth, cortical thickness; GM, grey matter; sNfL, serum neurofilament light chain.

Higher mean inflammatory sNfL level predicted lower total GM (standardised β=−0.399, p=0.040) and deep GM (standardised β=−0.556, p=0.010) volume, lower mean cortical thickness (standardised β=−0.581, p=0.010) and higher logT2LC (standardised β=0.498, p=0.018) (figure 2). Of all the clinical outcomes, higher mean inflammatory sNfL level was associated with a higher score (higher disability) on the logD9-HPT (standardised β=0.593, p=0.004) and a lower increase (less disability accumulation) in the logND9-HPT score (standardised β=−0.498, p=0.024) between month 24 and the 10-year follow-up.

Fraction of active MRI scans was not a significant predictor in any of the models (table 3).

#### Exploratory analyses

In a subset of patients, inflammatory sNfL levels were not associated with any MRI measurement obtained at the 10-year follow-up, after correcting for MRI atrophy measurements obtained at month 24 (online supplemental table 3).

Higher mean inflammatory sNfL levels and D9-HPT scores, but not ND9-HPT scores, were significantly associated with lower cortical thickness in the left and right precentral gyrus (online supplemental table 4).

### Mean non-inflammatory sNfL level

The effect of mean non-inflammatory sNfL level on MRI and clinical measures at the 10-year follow-up is shown in table 4. The mean non-inflammatory sNfL level was not associated with any of the MRI measures. For the clinical measures, higher levels were solely associated with a higher SDMT score (better attention score) at the 10-year follow-up (standardised β=0.473, p=0.003).

**Table 4:** Model 2: The association of mean non-inflammatory sNfL level with MRI atrophy and clinical measures at the 10-year follow-up, corrected for age, sex, DMT use, eTIV, BL T2LC and BL Gd+ LC*

MRI/clinical measure		Mean non-inflammatory sNfL level				Full model	
	N	B	Std. B	95% CI	P value†	R^2^ adj.	P value
Total GM volume	36				NS		
Total WM volume	36				NS		
Total deep GM volume	36				NS		
Thalamus volume	36				NS		
Mean Cth	36				NS		
logLesion volume‡	36				NS		
Lesion count	36				NS		
EDSS≥4§	40				NS		
logT25FW‡	38				NS		
logChange in T25FW‡	38				NS		
logD9-HPT‡	38				NS		
logChange in D9-HPT‡	38				NS		
logND9-HPT‡	37				NS		
logChange in ND9-HPT‡	37				NS		
PASAT	40				NS		
Change in PASAT	40				NS		
Oral SDMT	35	0.548	0.473	0.196 to 0.900	**0.003**	0.380	<0.001

*Non-significant covariates removed from final model by backward elimination.

†Adjusted p values after controlling the false discovery rate (FDR) for multiple hypothesis testing.

‡Dependent variable log transformed due to non-normality (log-linear transformation).

§Analysed by logistic regression.

adj, adjusted; B, beta; BL, baseline; Cth, cortical thickness; D9-HPT, dominant hand Nine-Hole Peg Test; DMT, disease modifying therapy; EDSS, Expanded Disability Status Scale; eTIV, estimated total intracranial volume; Gd+, gadolinium-enhancing; GM, grey matter; LC, lesion count; ND9-HPT, non-dominant hand Nine-Hole Peg Test; PASAT, Paced Auditory Serial Addition Test; SDMT, Symbol Digit Modalities Test; sNfL, serum neurofilament light chain; Std, standardised; T25FW, timed 25-foot walk; WM, white matter.

## Discussion

We found that higher mean sNfL level, measured over a 2-year period in patients with RRMS, was not associated with MRI or clinical measures after 10 years. However, when separately assessing mean sNfL levels measured during periods of active inflammation, higher levels associated significantly with lower total GM and deep GM volume, lower cortical thickness, higher T2 LC and higher disability measured by the D9-HPT. Lastly, sNfL levels during remission were not associated with long-term atrophy or disability progression. These findings suggest that sNfL levels during active inflammation may better predict atrophy and disability progression than overall mean sNfL and sNfL levels during remission.

Inflammatory sNfL levels were analysed in samples collected during periods with focal active inflammation, reflecting the extent of acute axonal damage.^9^ The association with GM atrophy measured after 10 years, implies that the delayed neurodegeneration in certain GM regions is at least partly secondary to focal inflammatory damage, most likely through anterograde or retrograde neuroaxonal degeneration along WM tracts.^28^ An alternative hypothesis could be that the association is based on pseudoatrophy following resolved inflammatory activity, but as pseudoatrophy is shown to mainly affect the WM,^29^ this seems less plausible. Elevated NfL levels predicting secondary neurodegeneration have been suggested in previous works,^17 30^ finding an association between higher sNfL levels and atrophy progression in deep GM over a 5-year^17^ and 6-year^19^ follow-up period. Our study supports this further, by assessing inflammatory sNfL levels separately and finding that the associated GM atrophy was located in deep GM and the cerebral cortex, areas known to be highly interconnected through various WM circuits,^31 32^ therefore susceptible to degradation.^33 34^ When correcting for MRI atrophy measures at month 24, associations with inflammatory sNfL levels were no longer significant. This may imply that the atrophy progression develops relatively early in the disease course. However, this exploratory analysis was conducted in only a subset of patients, and should in future works be repeated in larger cohorts.

We found that higher sNfL levels were associated with a higher score (higher disability) on the logD9-HPT. This result is partly in line with a previous study, showing that the patient group with prominent spinothalamic atrophy progression had higher sNfL levels and developed motor disability faster than the groups with atrophy progression in other regions.^19^ The difference between groups was most evident when assessing walking speed (T25FW) and finger dexterity (9-HPT).^19^ Hypothetically, the associated disability progression may result from acute disruption of crucial WM tracts (ie, the corticospinal tract) and secondary upstream neurodegeneration in connected GM areas (ie, the primary motor cortex).^34^ In our study, higher inflammatory sNfL levels were also associated with a lower change (less disability accumulation) in the logND9-HPT. While this finding does not coincide with the suggested hypothesis, the analysis may have been influenced by statistical power-issues and outliers. Furthermore, in an exploratory analysis (online supplemental table 4), lower cortical thickness in the precentral gyri was associated with both higher inflammatory sNfL levels and higher disability measured by the D9-HPT, but not by the ND9-HPT.

We found no associations between sNfL levels and EDSS. In previous research, the relation between sNfL and EDSS progression over 10 years or more is variable,^13 14 16^ suggested to be influenced by the difference in disease severity between cohorts.^10^ Our study of a limited number of patients, with relatively low overall disability progression (namely: up to EDSS 2.8), may be affected by the known low sensitivity to change in EDSS,^35^ especially for lower scores.

A higher fraction of MRI scans with new Gd+ lesions was not a significant predictor in any of the models. Compared with the results seen for inflammatory sNfL, this lack of significant associations may be due to the less sensitive fractional measure used, based on dichotomised values. Nevertheless, the discrepant results for the two predictors may also mean that future neurodegeneration and disability depend on the extent of axonal damage and location of an episode with a new Gd+ lesion(s), more than the frequency of such episodes.

Except for a positive relationship between non-inflammatory sNfL level and oral SDMT, none of the models for this predictor were significant. This may be influenced by statistical power issues and outliers, as the sample size was small (40 patients), with small overall variability in sNfL levels. As only patients with at least three samplings of non-inflammatory sNfL levels available were included, analyses may also be subject to selection bias, selecting patients with an overall less active disease course (none of the patients had at least three non-inflammatory and inflammatory measurements available). However, repeating the analyses including patients with a minimum of two non-inflammatory sNfL levels, and subsequently patients with periods of both remission and active disease (35 patients), yielded the same results. The results are also in line with a recent study finding no association between NfL level and disease progression in natalizumab-treated patients, after correcting for MRI activity.^12^ From these findings, the authors hypothesised that the sensitivity of NfL is too low to capture more subtle neuroaxonal damage not associated with active inflammation.

The findings in our study may have clinical relevance. Long-term outcomes were independently predicted by sNfL levels during inflammatory episodes, and not by the frequency of such episodes during the first 2 years. Hence, measuring sNfL levels during relapses may be a way to quantify the extent of ongoing axonal damage, possibly indicating the risk of permanent disability, either caused by direct axonal damage during active inflammation, or by the delayed secondary neurodegenerative process affecting GM in connected regions. This added information may support clinicians in subsequent monitoring and treatment decisions. Furthermore, the addition of sNfL to treatment response scoring tools^36 37^ could possibly increase their predictive value, and should be assessed in future studies.

Correcting for DMT use did not change the associations between sNfL levels and long-term outcomes. However, use of high efficiency therapies (indicating disease activity) over the follow-up was independently associated with disability accumulation measured by the 9-HPT (results not shown). As patients were treated similarly until the conclusion of the OFAMS Study (first treatment naïve, then treated with interferons), this suggests that potent treatment during the first years after diagnosis is important for long-term prognosis, especially in patients with high disease activity.

This study has limitations, the main challenges and suggestions for future research are summarised in table 5. There is a degree of uncertainty in defining sNfL levels as ‘inflammatory’ or ‘non-inflammatory’. Regarding different lesion types, we focused on their relation with Gd+ lesions, as these are strongly associated with active inflammation and NfL release,^10^ and can be temporally identified with great certainty. However, at BL, month 12 and 24, there was no MRI scan available from the previous months to decide on recent inflammatory activity, and spinal lesions were not accounted for. After a Gd+ lesion, increased sNfL levels may persist for up to 90 days,^9^ and a previous study on this patient cohort found elevated sNfL levels up to 1 month before and 2 months after the appearance of Gd+ lesions,^23^ indicating that the windows for defining a sNfL measurement as inflammatory or non-inflammatory in the current study may be too narrow and too wide, respectively. Non-inflammatory measurements are at highest risk of misclassification, ideally collected with a wider interval between new lesions, to ensure the levels are not influenced by inflammatory damage. These considerations underline the need to clarify the relationship between the temporal dynamics of NfL levels and the evolution of lesions. With the available data in this cohort, our definitions were set to maximise the contrast between inflammatory and non-inflammatory periods, while still maintaining an acceptable group size. Despite these uncertainties, the associations with long-term outcomes found in this study were clearly different between the two measurements, substantiating the sensitivity of the set definitions. Moreover, the patterns of significant associations were similar when analysing mean inflammatory and non-inflammatory sNfL levels calculated from only two or more measurements, also including patients (35 patients) with both inflammatory and non-inflammatory sNfL levels during the 2-year follow-up.

**Table 5:** Current research challenges and suggestions for future research

Research challenges	Suggestions for future research
Clarify the temporal relation between sNfL levels and new, enlarging and diminishing lesions, for example, T2 hyperintense lesions T1 hypointense lesions T1 Gd+ hyperintense lesions GM lesions Spinal lesions	Prospective studies Sufficient sample size Extensive follow-up time Frequent follow-up visits, including: Imaging techniques suited for analyses of longitudinal lesion and atrophy progression. Statistical analyses correcting for known risk factors and modulators of disease progression: Baseline and on-study lesion activity. Previous and on-study therapeutic interventions. Genetic and environmental risk factors. Comorbid conditions. Consider using z scores for sNfL derived from a healthy control group or a reference database.^26^.
Clarify the temporal relation between sNfL levels and GM atrophy progression, for example, Global brain GM atrophy Regional brain GM atrophy Spinal atrophy	
Clarify the value of sNfL as an independent predictive biomarker of long-term prognosis.	
Establishing sNfL reference values.	

Gd+, gadolinium-enhancing; GM, grey matter; sNfL, serum neurofilament light chain.

GM volumes were measured cross-sectionally from data collected at the 10-year follow-up visit and month 24, limiting our ability to conclude on longitudinal atrophy progression. When correcting for atrophy measures obtained at month 24, the associations with GM atrophy after 10 years were no longer significant. This analysis may have been underpowered due to the small sample size, so further investigations in larger patient populations, with regular and more frequent follow-up visits, may clarify the temporal relation between inflammatory WM damage, sNfL levels and GM atrophy. Additionally, future studies should consider the effect of lesion volume and lesion volume change, preferably over longer time periods. In this study, we corrected for Gd+ and T2 LC at BL, as we deemed BL volume measures too unreliable to include, due to the quality of the MRI data (eg, partial brain coverage, large slice thickness, 2D images).

Lastly, atrophy measurements were obtained from postcontrast images, which is not the standard approach for FreeSurfer. However, recent work has shown excellent consistency between values obtained from precontrast and postcontrast images.^24^

### Conclusion

Higher sNfL levels during early periods of active inflammation, but probably not during remission, in patients with RRMS predicted GM atrophy and specific aspects of clinical disability 10 years later. The findings suggest that subsequent long-term GM atrophy is mainly due to neuroaxonal degradation induced by acute inflammation.

## Data Availability

Data are available upon reasonable request.

## References

[1] Kutzelnigg A , Lucchinetti CF , Stadelmann C , et al . Cortical demyelination and diffuse white matter injury in multiple sclerosis. Brain 2005;128:2705–12. 10.1093/brain/awh641 16230320

[2] Charil A , Zijdenbos AP , Taylor J , et al . Statistical mapping analysis of lesion location and neurological disability in multiple sclerosis: application to 452 patient data sets. Neuroimage 2003;19:532–44. 10.1016/S1053-8119(03)00117-4 12880785

[3] Steenwijk MD , Geurts JJG , Daams M , et al . Cortical atrophy patterns in multiple sclerosis are non-random and clinically relevant. Brain 2016;139:115–26. 10.1093/brain/awv337 26637488

[4] Ceccarelli A , Rocca MA , Pagani E , et al . A voxel-based morphometry study of grey matter loss in MS patients with different clinical phenotypes. Neuroimage 2008;42:315–22. 10.1016/j.neuroimage.2008.04.173 18501636

[5] Comabella M , Montalban X . Body fluid biomarkers in multiple sclerosis. Lancet Neurol 2014;13:113–26. 10.1016/S1474-4422(13)70233-3 24331797

[6] Teunissen CE , Khalil M . Neurofilaments as biomarkers in multiple sclerosis. Mult Scler 2012;18:552–6. 10.1177/1352458512443092 22492131

[7] Bielekova B , McDermott MP . Will CSF biomarkers guide future therapeutic decisions in multiple sclerosis? Neurology 2015;84:1620–1. 10.1212/WNL.0000000000001506 25809305

[8] Kuhle J , Barro C , Disanto G , et al . Serum neurofilament light chain in early relapsing remitting MS is increased and correlates with CSF levels and with MRI measures of disease severity. Mult Scler 2016;22:1550–9. 10.1177/1352458515623365 26754800

[9] Rosso M , Gonzalez CT , Healy BC , et al . Temporal association of sNfL and gad-enhancing lesions in multiple sclerosis. Ann Clin Transl Neurol 2020;7:945–55. 10.1002/acn3.51060 32452160 PMC7318095

[10] Bittner S , Oh J , Havrdová EK , et al . The potential of serum neurofilament as biomarker for multiple sclerosis. Brain 2021;144:2954–63. 10.1093/brain/awab241 34180982 PMC8634125

[11] Khalil M . Are neurofilaments valuable biomarkers for long-term disease prognostication in MS? Mult Scler 2018;24:1270–1. 10.1177/1352458518791518 30066596

[12] Bridel C , Leurs CE , van Lierop ZYGJ , et al . Serum neurofilament light association with progression in natalizumab-treated patients with relapsing-remitting multiple sclerosis. Neurology 2021;97:e1898–905. 10.1212/WNL.0000000000012752 34504023

[13] Cantó E , Barro C , Zhao C , et al . Association between serum neurofilament light chain levels and long-term disease course among patients with multiple sclerosis followed up for 12 years. JAMA Neurol 2019;76:1359–66. 10.1001/jamaneurol.2019.2137 31403661 PMC6692664

[14] Thebault S , Abdoli M , Fereshtehnejad S-M , et al . Serum neurofilament light chain predicts long term clinical outcomes in multiple sclerosis. Sci Rep 2020;10:10381. 10.1038/s41598-020-67504-6 32587320 PMC7316736

[15] Barro C , Benkert P , Disanto G , et al . Serum neurofilament as a predictor of disease worsening and brain and spinal cord atrophy in multiple sclerosis. Brain 2018;141:2382–91. 10.1093/brain/awy154 29860296

[16] Chitnis T , Gonzalez C , Healy BC , et al . Neurofilament light chain serum levels correlate with 10-year MRI outcomes in multiple sclerosis. Ann Clin Transl Neurol 2018;5:1478–91. 10.1002/acn3.638 30564615 PMC6292183

[17] Jakimovski D , Kuhle J , Ramanathan M , et al . Serum neurofilament light chain levels associations with gray matter pathology: a 5-year longitudinal study. Ann Clin Transl Neurol 2019;6:1757–70. 10.1002/acn3.50872 31437387 PMC6764487

[18] Filippi P , Vestenická V , Siarnik P , et al . Neurofilament light chain and MRI volume parameters as markers of neurodegeneration in multiple sclerosis. Neuro Endocrinol Lett 2020;41:17–26. 32338853

[19] Tsagkas C , Parmar K , Pezold S , et al . Classification of multiple sclerosis based on patterns of CNS regional atrophy covariance. Hum Brain Mapp 2021;42:2399–415. 10.1002/hbm.25375 33624390 PMC8090784

[20] Malmeström C , Haghighi S , Rosengren L , et al . Neurofilament light protein and glial fibrillary acidic protein as biological markers in MS. Neurology 2003;61:1720–5. 10.1212/01.WNL.0000098880.19793.B6 14694036

[21] Torkildsen O , Wergeland S , Bakke S , et al . ω-3 fatty acid treatment in multiple sclerosis (OFAMS Study): a randomized, double-blind, placebo-controlled trial. Arch Neurol 2012;69:1044–51. 10.1001/archneurol.2012.283 22507886

[22] Wesnes K , Myhr K-M , Riise T , et al . Low vitamin D, but not tobacco use or high BMI, is associated with long-term disability progression in multiple sclerosis. Mult Scler Relat Disord 2021;50:102801. 10.1016/j.msard.2021.102801 33636616

[23] Varhaug KN , Barro C , Bjørnevik K , et al . Neurofilament light chain predicts disease activity in relapsing-remitting MS. Neurol Neuroimmunol Neuroinflamm 2018;5:e422. 10.1212/NXI.0000000000000422 29209636 PMC5707445

[24] Lie IA , Kerklingh E , Wesnes K , et al . The effect of gadolinium-based contrast-agents on automated brain atrophy measurements by FreeSurfer in patients with multiple sclerosis. Eur Radiol 2022;32:3576–87. 10.1007/s00330-021-08405-8 34978580 PMC9038813

[25] Schmidt P , Gaser C , Arsic M , et al . An automated tool for detection of FLAIR-hyperintense white-matter lesions in multiple sclerosis. Neuroimage 2012;59:3774–83. 10.1016/j.neuroimage.2011.11.032 22119648

[26] Benkert P , Meier S , Schaedelin S , et al . Serum neurofilament light chain for individual prognostication of disease activity in people with multiple sclerosis: a retrospective modelling and validation study. Lancet Neurol 2022;21:246–57. 10.1016/S1474-4422(22)00009-6 35182510

[27] Benjamini Y , Hochberg Y . Controlling the false discovery rate: a practical and powerful approach to multiple testing. J R Stat Soc Series B Stat Methodol 1995;57:289–300. 10.1111/j.2517-6161.1995.tb02031.x

[28] Dendrou CA , Fugger L , Friese MA . Immunopathology of multiple sclerosis. Nat Rev Immunol 2015;15:545–58. 10.1038/nri3871 26250739

[29] Vidal-Jordana A , Sastre-Garriga J , Pérez-Miralles F , et al . Early brain pseudoatrophy while on natalizumab therapy is due to white matter volume changes. Mult Scler 2013;19:1175–81. 10.1177/1352458512473190 23319072

[30] Srpova B , Uher T , Hrnciarova T , et al . Serum neurofilament light chain reflects inflammation-driven neurodegeneration and predicts delayed brain volume loss in early stage of multiple sclerosis. Mult Scler 2021;27:52–60. 10.1177/1352458519901272 31961243

[31] Sherman SM . Functioning of circuits connecting thalamus and cortex. Compr Physiol 2017;7:713–39. 10.1002/cphy.c160032 28333385

[32] Hasan KM , Kamali A , Kramer LA . Mapping the human brain white matter tracts relative to cortical and deep gray matter using diffusion tensor imaging at high spatial resolution. Magn Reson Imaging 2009;27:631–6. 10.1016/j.mri.2008.10.007 19128910

[33] Henry RG , Shieh M , Amirbekian B , et al . Connecting white matter injury and thalamic atrophy in clinically isolated syndromes. J Neurol Sci 2009;282:61–6. 10.1016/j.jns.2009.02.379 19394969

[34] Bergsland N , Laganà MM , Tavazzi E , et al . Corticospinal tract integrity is related to primary motor cortex thinning in relapsing-remitting multiple sclerosis. Mult Scler 2015;21:1771–80. 10.1177/1352458515576985 25791368

[35] Meyer-Moock S , Feng Y-S , Maeurer M , et al . Systematic literature review and validity evaluation of the expanded disability status scale (EDSS) and the multiple sclerosis functional composite (MSFC) in patients with multiple sclerosis. BMC Neurol 2014;14:58. 10.1186/1471-2377-14-58 24666846 PMC3986942

[36] Río J , Castilló J , Rovira A , et al . Measures in the first year of therapy predict the response to interferon beta in MS. Mult Scler 2009;15:848–53. 10.1177/1352458509104591 19542263

[37] Sormani MP , Freedman MS , Aldridge J , et al . MAGNIMS score predicts long-term clinical disease activity-free status and confirmed disability progression in patients treated with subcutaneous interferon beta-1a. Mult Scler Relat Disord 2021;49:102790. 10.1016/j.msard.2021.102790 33571946

