# Supplementary material for: Serum neurofilament as a predictor of 10-year grey matter atrophy and clinical disability in multiple sclerosis: a longitudinal study
Source: J Neurol Neurosurg Psychiatry. 2022 Jun 1;93(8):849–57. doi: 10.1136/jnnp-2021-328568 (PMC9304101; doi:10.1136/jnnp-2021-328568)
Supplement: Supplementary data [file jnnp-2021-328568supp001.pdf]

Appendix 1

Lesion segmentation. Lesion segmentation was done on FLAIR images using lesion segmentation tool (LST) (version 2.0.15; http://applied-statistics.de/lst.html)[1]. To optimise lesion filling, gadolinium-enhancing regions (both lesions and other regions) were first removed, by applying an upper intensity threshold at the 98th percentile. Next, the FMRIB Software Library (FSL) (version 5.0.10; http://www.fmrib.ox.ac.uk/fsl) was used to fill in abnormal voxels in these preprocessed T1-weighted images using the lesion_filling tool[2]. Then only the filled lesion voxels were pasted back into the original post-contrast 3D T1-weighted images to create the final lesion filled images.

Morphological reconstruction. Cortical reconstruction and parcellation for (local) cortical volume and thickness measurement and subcortical segmentation were performed with FreeSurfer version 7.1.1, a freely available software package for academic use, available through online download (http://surfer.nmr.mgh.harvard.edu/). The technical details of FreeSurfer procedures have been previously described[3, 4].

Quality control was performed by visual inspection, discarding cases with large segmentation errors. In cases where only specific anatomical regions were incorrectly segmented, we chose not to apply any manual corrections for these errors in our analyses.

The Desikan-Killiany atlas[5] was used to extract cortical thickness measures (mean cortical thickness, left and right hemisphere). Furthermore, total cerebral GM and WM volume, total deep GM and thalamus volume (left and right hemisphere) were obtained. Because of frequent suboptimal segmentation of the temporal pole, this region was excluded when calculating the total GM volume.

1. Schmidt P, Gaser C, Arsic M, et al. An automated tool for detection of FLAIR-hyperintense white-matter lesions in Multiple Sclerosis. *NeuroImage* 2012;59(4):3774-83.
2. Battaglini M, Jenkinson M, De Stefano N. Evaluating and reducing the impact of white matter lesions on brain volume measurements. *Hum Brain Mapp* 2012;33(9):2062-71.
3. Fischl B. FreeSurfer. *NeuroImage* 2012;62(2):774-81.
4. Dale AM, Fischl B, Sereno MI. Cortical surface-based analysis. I. Segmentation and surface reconstruction. *NeuroImage* 1999;9(2):179-94.
5. Desikan RS, Ségonne F, Fischl B, et al. An automated labeling system for subdividing the human cerebral cortex on MRI scans into gyral based regions of interest. *NeuroImage* 2006;31(3):968-80.
