# Supplementary material for: Serum neurofilament as a predictor of 10-year grey matter atrophy and clinical disability in multiple sclerosis: a longitudinal study
Source: J Neurol Neurosurg Psychiatry. 2022 Jun 1;93(8):849–57. doi: 10.1136/jnnp-2021-328568 (PMC9304101; doi:10.1136/jnnp-2021-328568)
Supplement: Supplementary data [file jnnp-2021-328568supp002.pdf]

Supplemental material

Supplemental Table 1. The association of mean inflammatory sNfL level calculated from at least two measurements and fGd+ with MRI atrophy and clinical measures at the 10-year follow-up, corrected for age, sex, DMT use, eTIV (MRI volume measures), BL T2LC and BL Gd+LC.

Supplemental Table 1. Model 1: The association of inflammatory sNfL level and fGd+ with MRI atrophy and clinical measures at the 10-year follow-up, corrected for age, sex, DMT use, eTIV, BL T2LC and BL Gd+LC[a].

		**Mean inflammatory sNfL level**				**fGd+**				**Full model**	
MRI/clinical measure	*N*	*B*	*Std. B*	*95% conf. interval*	*p-value**	*B*	*Std. B*	*95% conf. interval*	*p-value**	R^2 *adj.*	*p-value*
Total GM volume	35				NS				NS		
Total WM volume	35				NS				NS		
Total subcortical GM volume	35	-96.27	-0.397	-175.064, -17.482	**0.036**				NS	0.132	0.018
Thalamus volume	35				NS				NS		
Mean Cth	35	-0.002	-0.433	-0.004, -0.001	**0.036**				NS	0.163	0.009
logLesion volume[b]	39				NS				NS		
logLesion count[b]	39	0.004	0.508	0.002, 0.007	**0.008**				NS	0.238	0.001
EDSS≥4[c]	45				NS				NS		
logT25FW[b]	43				NS				NS		
logChange in T25FW[b]	41				NS				NS		
logD9-HPT[b]	43				NS				NS		
logChange in D9-HPT[b]	41				NS	-0.554	-0.406	-0.928, -0.180	**0.010**	0.285	0.004

logND9-HPT[b]	42				NS				NS		
logChange in ND9-HPT[b]	41	-0.454	-0.320	-0.803, -0.105	**0.012**	-0.005	-0.405	-0.009, -0.001	**0.035**	0.367	0.001
PASAT	42				NS				NS		
Change in PASAT	40				NS				NS		
Oral SDMT	42				NS				NS		

[a] *Non-significant covariates removed from final model by backward elimination.*

[b] *Dependent variable log transformed due to non-normality (log-linear transformation).*

[c] *Analysed by logistic regression.*

**Adjusted p-values after controlling the False discovery rate (FDR) for multiple hypothesis testing.*

Abbreviations: sNfL=serum neurofilament light chain, eTIV=estimated total intracranial volume, DMT=disease modifying therapy, BL=baseline, Gd+=gadolinium-enhancing, LC=lesion count, fGd+=fraction of MRI scans with new Gadolinium-enhancing lesion, N=number, B=beta, Std=standardised, GM=grey matter, WM=white matter, NS=not significant, Cth=cortical thickness, EDSS=expanded disability status scale, T25FW=timed 25-foot walk, D9-HPT=dominant hand 9-hole peg test, ND9-HPT=non-dominant hand 9-hole peg test, PASAT=paced auditory serial addition test, SDMT=symbol digit modalities test.

Supplemental Table 2. The association of mean non-inflammatory sNfL level calculated from at least two measurements with MRI atrophy and clinical measures at the 10-year follow-up, corrected for age, sex, DMT use, eTIV (MRI volume measures), BL T2LC and BL Gd+LC.

Supplemental Table 2. Model 2: The association of mean non-inflammatory sNfL level with MRI atrophy and clinical measures at the 10-year follow-up, corrected for age, sex, DMT use, eTIV, BL T2LC and BL Gd+LC[a].

		**Mean non-inflammatory sNfL level**				**Full model**	
MRI/ clinical measure	*N*	*B*	*Std. B*	*95% conf. interval*	*p-value**	*R^2 adj.*	*p-value*
Total GM volume	59				NS		
Total WM volume	59				NS		
Total deep GM volume	59				NS		
Thalamus volume	59				NS		
Mean Cth	59				NS		
logLesion volume[b]	60				NS		
Lesion count	60				NS		
EDSS≥4[c]	67				NS		
logT25FW[b]	64				NS		
logChange in T25FW[b]	62				NS		
logD9-HPT[b]	63				NS		
logChange D9-HPT[b]	61				NS		
logND9-HPT[b]	62				NS		
Change in ND9-HPT	60				NS		
PASAT	65				NS		
Change in PASAT	63				NS		
Oral SDMT	60	0.446	0.389	0.168, 0.724	**0.002**	0.321	<0.001

[a] *Non-significant covariates removed from final model by backward elimination.*

[b] *Dependent variable log transformed due to non-normality (log-linear transformation).*

[c] *Analysed by logistic regression.*

**Adjusted p-values after controlling the False discovery rate (FDR) for multiple hypothesis testing.*

Abbreviations: sNfL=serum neurofilament light chain, eTIV=estimated total intracranial volume, DMT=disease modifying therapy, BL=baseline, Gd+=gadolinium-enhancing, LC=lesion count, N=number, B=beta, Std=standardised, GM=grey matter, WM=white matter, NS=not significant, Cth=cortical thickness, EDSS=expanded disability status scale, T25FW=timed 25-foot walk, D9-HPT=dominant hand 9-hole peg test, ND9-HPT=non-dominant hand 9-hole peg test, PASAT=paced auditory serial addition test, SDMT=symbol digit modalities test.

Supplemental table 3. The association of mean inflammatory sNfL level calculated from at least three measurements and fGd+ with MRI atrophy and clinical measures at the 10-year follow-up, corrected for age, sex, DMT use, eTIV (MRI volume measures), BL T2LC, BL Gd+LC and MRI atrophy measurements obtained at month 24.

Supplemental table 3. The association of inflammatory sNfL level and fGd+ with MRI atrophy measurements at the 10-year follow-up, corrected for age, sex, DMT use, eTIV, BL T2LC, Gd+LC, and MRI measurements at month 24[a]

		**Mean inflammatory sNfL level**				**fGd+**				**Full model**	
MRI measure	*N*	*B*	*Std. B*	*95% conf. interval*	*p-value**	*B*	*Std. B*	*95% conf. interval*	*p-value**	R^2 *adj.*	*p-value*
Total GM volume	19				NS				NS		
Total WM volume	19				NS				NS		
Total deep GM volume	19				NS				NS		
Thalamus volume	19				NS				NS		
Mean Cth	19				NS				NS		

[a] *Non-significant covariates removed from final model by backward elimination.*

**Adjusted p-values after controlling the False discovery rate (FDR) for multiple hypothesis testing.*

Abbreviations: sNfL=serum neurofilament light chain, eTIV=estimated total intracranial volume, DMT=disease modifying therapy, BL=baseline, Gd+=gadolinium-enhancing, LC=lesion count, fGd+=fraction of MRI scans with new Gadolinium-enhancing lesion, N=number, B=beta, Std=standardised, GM=grey matter, WM=white matter, NS=not significant, Cth=cortical thickness.

Supplemental Table 4. Multiple linear regression with cortical thickness of the left and right precentral gyrus as dependent variables, mean inflammatory sNfL level, D9-HPT, change in D9-HPT, ND9-HPT and change in ND9-HPT are included as independent variables in separate models, all corrected for age, sex, DMT use, BL T2LC, BL Gd+LC and fGd+.

Supplemental Table 4. The association of mean inflammatory sNfL level, D9-HPT and ND9-HPT with mean cortical thickness of the precentral gyri at the 10-year follow-up, corrected for age, sex, DMT use, BL T2LC, BL Gd+LC and fGd+[a].

Independent variable	*N*	*B*	*Std. B*	*95% conf. interval*	*p-value**	*R^2 adj.*	*p-value*
**Cortical thickness left precentral gyrus**							
Mean inflammatory sNfL level	25	-0.006	-0.498	-0.010, -0.001	**0.015**	0.216	0.011
D9-HPT	66	-0.011	-0.426	-0.018, -0.005	**0.001**	0.169	<0.001
Change in D9-HPT	62				NS		
ND9-HPT	64				NS		
Change in ND9-HPT	62				NS		
**Cortical thickness right precentral gyrus**							
Mean inflammatory sNfL level	25	-0.006	-0.488	-0.010, -0.001	**0.013**	0.206	0.013
D9-HPT	66	-0.010	-0.410	-0.016, -0.005	**0.001**	0.276	<0.001
Change in D9-HPT	62				NS		
ND9-HPT	64				NS		
Change in ND9-HPT	62				NS		

[a] Non-significant covariates removed from final model by backward elimination.

**Adjusted p-values after controlling the False discovery rate (FDR) for multiple hypothesis testing.*

Abbreviations: sNfL=serum neurofilament light, BL=baseline, Gd+=gadolinium-enhancing, LC=lesion count, fGd+=fraction of MRI scans with new Gadolinium-enhancing lesion, D9-HPT=dominant hand 9-hole peg test, ND9-HPT=non-dominant hand 9-hole peg test, DMT=disease modifying therapy, N=number, B=beta, Std=standardised, conf=confidence, adj=adjusted.
